# Small Bowel Endometriosis; A Case Report and Review of Literature

**DOI:** 10.1016/j.amsu.2019.09.005

**Published:** 2019-09-18

**Authors:** Haytham Alabbas, Tariq Awidah, Obada Alhallaq, Tamir Babiker

**Affiliations:** International Medical Center, Jeddah, Saudi Arabia

**Keywords:** Case report, Small bowel, Endometriosis, Terminal ileum, Bowel obstruction, Bowel resection

## Abstract

• Small bowel obstruction has a broad list of diagnosis.
• Thorough history and physical examination along with proper imaging is the key in determining the cause.
• Endometriosis if diagnosed preoperatively may respond to hormonal therapy.
• Laparoscopic exploration may be both diagnostic and therapeutic.
• Small Bowel Endometriosis; A Case Report and Review of Literature.

## 1 Introduction

Endometriosis usually occurs in menstruating women up to 15% [1]. Most common gastrointestinal involvement of endometriosis is found in the sigmoid colon, rectum and terminal ileum in 3%–37% of women [2]. Proliferation and infiltration of the intestinal wall with endometrial implants may cause fibrotic reaction with formation of strictures and adhesions, probably from the effect of cyclical hormonal influences of menstruation. Eventually, this may lead to bowel obstruction and recurrent abdominal pain [3]. We are presenting an interesting case of terminal ileum endometriosis required surgical resection.

## 2 Methods

This is a case reported in line with SCARE criteria [4].

### 2.1 Case presentation

**Fig. 1:**
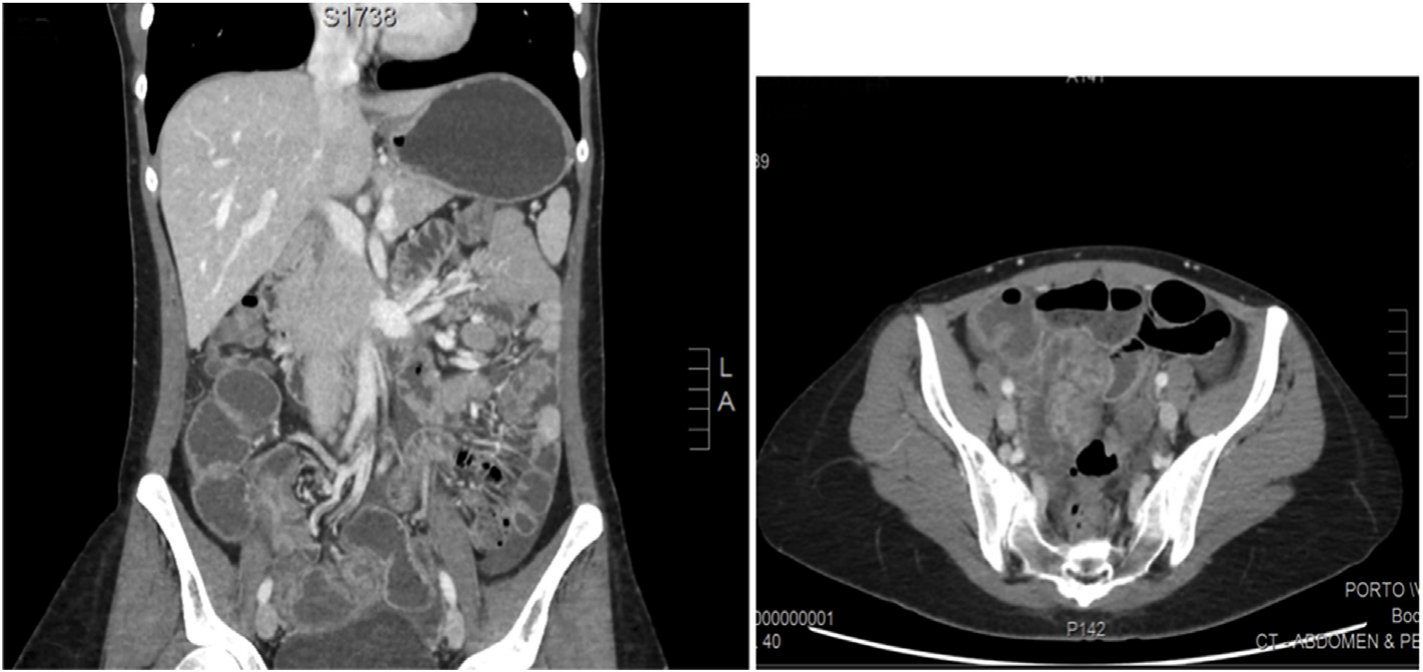
CT scan of the abdomen showing dilatation of the terminal ileum due to a compressing effect by mass causing significant stenosis and partial small bowel obstruction.

**Fig. 2:**
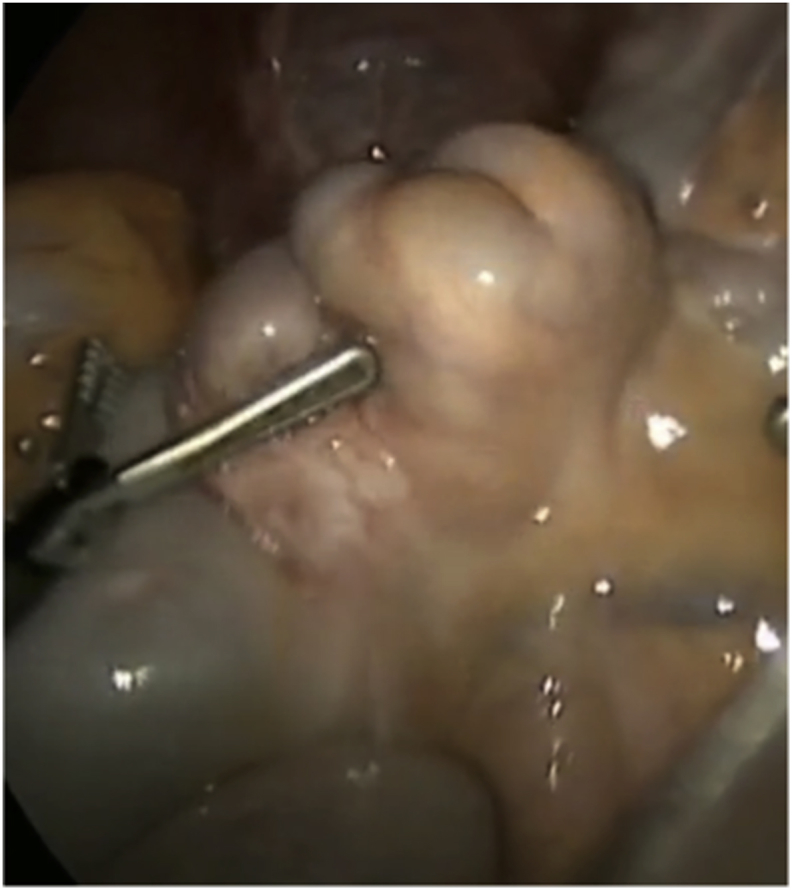
Irregular mass attached to the serosa of the terminal ileum.

This patient has been suffering from a long standing undiagnosed abdominal pain that started eight years earlier. The pain was colicky, localized in the right iliac fossa and seldom associated with nausea and vomiting. The laboratories evaluations were normal. Few years back, patient underwent ultrasound of the abdomen for work up, showed a right ovarian complex cyst. Despite removing the ovarian cyst laparoscopically, patient still had persistent pain. Patient was referred to gastroenterology and underwent upper and lower gastrointestinal endoscopies for suspicion of Crohn's disease. These endoscopic evaluations and biopsies were normal. Patient presented to our emergency room with abdominal pain, nausea, vomiting and constipation, but passing flatus for 2 days. She was vitally stable. Her abdomen was distended with tenderness over the right lower quadrant but no peritoneal signs. Her abdominal series showed multiple air-fluids levels, dilated segments of small bowel and absence of gas in the rectum. Patient was resuscitated and an enhanced CT scan with contrast was done to determine the nature of the obstruction (Fig. 1). There was a suspicion for terminal ileum intussusceptions that was planned for surgical intervention but clinically, patient improved. Patient responded well to conservative management and discharged home three days later on oral diet. She came for follow up but had similar complaints. Due to her recurrent semi-obstructive symptoms, she was scheduled for elective laparoscopic exploration. An irregular mass of 3 × 2cm was attached to the serosa of the terminal ileum. Intra-operative frozen section of the mass was sent (Fig. 2). Giving its close proximity to the ileocecal valve, decision was made to proceed with right hemicolectomy as discussed previously with patient.

## 3 Discussion

Endometriosis is characterized by the presence of functional endometrial tissue consisting of glands and stroma outside the uterus. Intestinal obstruction could be the presentation of ilial involvement. However, this is very rare up to 23% of all cases with ileum involvement [1]. Sampson's retrograde menstruation theory is the most widely accepted theory. Endometrial tissue refluxes through the fallopian tubes, implanting on the serosal surface of abdominal and pelvic organs which commonly occurs during menstruation [5]. However, other theories and factors, immunological, genetic and familial, could be involved in the pathogenesis of this disease [6]. Endometriosis presents usually with pelvic pain, infertility and dyspareunia [7], but it may often be non-specific. Because of terminal ileum involvement, our patient had recurrent pelvic pain with associated nausea and vomiting as a picture of partial small bowel obstruction.

Our patient is not married and her symptoms were not associated with menses and relapsed irregularly. Many GI diseases including small bowel obstruction, inflammatory bowel disease and neoplasm make preoperative diagnosis more elusive due to clinical similarities [7]. Relapsing symptoms are the hallmark of this disease [7]. Endometriosis of the distal ileum is an infrequent cause of intestinal obstruction, ranging from 7% to 23% of all cases with intestinal involvement [8]. The incidence of intestinal resection for bowel obstruction is 0.7% among patients undergone surgical treatment for abdomino-pelvic endometriosis [9]. However, endometriosis of the small bowel should be suspected in young, nulliparous patients with abdominal pain, in conjunction with signs of obstruction as this is the case in our patient [3].

Imaging work up may be inconclusive. Endoscopy and barium enema are not helpful because the disease does not involve the mucosa. In our patient, endoscopies and biopsies failed to show any inflammatory or neoplastic disease. However, Magnetic Resonance Imaging seems to have a higher sensitivity [10] and laparoscopy is considered gold standard. The diagnosis can be confirmed only on histology. Gastrointestinal endometriosis is usually found as an incidental finding on abdominal exploration. Asymptomatic and mildly symptomatic cases may be treated using hormonal treatment.

Suspicion of malignancy as well as acute obstructive cases may warrant a radical resection [7]. The management should include hormonal therapy and surgery. The former treatment with danazol or gonadotrophin-releasing hormone analogs may be used in patients without obstruction. However, resection of the involved bowel remains the choice of treatment for complicated or unresolved cases [11]. In our case, our patient had a prolonged history of relapsing symptoms for years with picture of recurrent partial small bowel obstruction in addition to an image showing possible mass causing small bowel stenosis and obstruction. Due to those reasons, we elected to perform diagnostic laparoscopy and found this endometrioma and performed right hemicolectomy after confirming frozen section with histopathology. Postoperatively, she has fully recovered with no abdominal complain. Her follow up visits up to two years showed resolution of her symptom.

## 4 Conclusion

Small bowel endometriosis is a rare entity. Clinical picture is similar to other GI diseases and careful assessment is warranted. Surgical resection is sometimes indicated for diagnosis or failing medical management. High suspicious of index is warranted in these cases.

## Ethical approval

Yes, our case report got ethical approval from International Medical Canter Ethical Committee. Reference number IMC-IRB # 2018-02-085.

## Sources of funding

The authors received no financial support for the research, authorship, and/or publication of this article.

## Author contribution

Author 1: Tamir Babkir.

Performed the procedure and supervised the literature review and the case report.

Author 2: Haytham Al-abbas.

Assisted the primary surgeon in the procedure, helped obtained the consent from patient, supervised the data collection, and review and summary of the manuscript.

Author 3: Tariq Awidah.

Wrote the paper.

Author 4: Obadah Alhallag.

Summarized the case and obtained the consent from patient.

## Conflicts of interest

The authors declare that there is no conflict of interest.

## Trial registry number

none.

## Provenance and peer review

Not commissioned, externally peer reviewed.

## Guarantor

Haytham Alabbas.

## Consent

Written informed consent was obtained from the patient for publication of this case report and accompanying images. A copy of the written consent is available for review by the Editor-in-Chief of this journal on request.
